# Investigating genomic medicine practice and perceptions amongst Australian non-genetics physicians to inform education and implementation

**DOI:** 10.1038/s41525-023-00360-1

**Published:** 2023-06-24

**Authors:** Amy Nisselle, Emily King, Bronwyn Terrill, Belinda Davey, Belinda McClaren, Kate Dunlop, Debra Graves, Sylvia Metcalfe, Clara Gaff

**Affiliations:** 1 Australian Genomics Health Alliance, Melbourne, VIC Australia; 2 Genomics in Society, Murdoch Children’s Research Institute, Melbourne, VIC Australia; 3 Department of Paediatrics, The University of Melbourne, Melbourne, VIC Australia; 4 Garvan Institute of Medical Research, Darlinghurst, NSW Australia; 5 School of Clinical Medicine, Faculty of Medicine and Health, UNSW Sydney, Sydney, NSW Australia; 6 School of Psychological Sciences, Monash University, Clayton, VIC Australia; 7 The Daffodil Centre, The University of Sydney, a joint venture with Cancer Council NSW, Sydney, NSW Australia; 8 Royal College of Pathologists of Australasia, Surry Hills, NSW Australia

**Keywords:** Medical genomics, Genetic testing, Health policy, Translational research, Outcomes research

## Abstract

Genomic medicine is being implemented on a global scale, requiring a genomic-competent health workforce. To inform education as part of implementation strategies to optimize adoption of genomics by non-genetics physicians, we investigated current practices, perceptions and preferences relating to genomic testing and education. Australian non-genetics physicians completed an online survey; we conducted univariate and multivariate analyses of determinants of confidence and engagement with genomic medicine. Confident or engaged respondents were more likely to be pediatricians, have completed continuing genomics education (CGE) and/or have genomics research experience. Confident or engaged respondents were also more likely to prefer to request genomic testing with support from genetics services than other models. Respondents who had completed CGE and were engaged reported higher confidence than those who were not engaged. We propose a progression of genomic competence aligned with service delivery models, where education is one enabler of mastery or independence to facilitate genomic tests (from referral to requesting with or without clinical genetics support). Workplace learning could provide additional impetus for adoption.

## Introduction

Genomic medicine is being implemented rapidly on a global scale^1^, with more than 65 international initiatives catalogued in 2020^2^. In Australia alone, more than a million genetics or genomics-related tests are performed each year, with just 7% requested by clinical geneticists^3^. The growth in genomic medicine is placing increasing demand internationally on the specialist genetics workforce^4^. The most common barrier to implementing large-scale genomic programs is a lack of genomics education and skill in the broader health workforce^5^, strengthening the recognized need for a broader genomic-competent health workforce^6,7^.

Adoption of genomic testing by physicians who are not specialists in genetics (‘non-genetics specialists’) has commenced. In 2011, 13% of American physicians reported having ordered a pharmacogenomic test^8^. In 2015, 5% of international neurologists reported ordering whole genome tests and 12% whole exome tests^9^. Some more recent studies suggest use of genomics by physicians, including medical oncologists, cardiologists and nephrologists, may be increasing^10–15^, but many are yet to incorporate genomics into their clinical practice.

Non-genetics specialists anticipate that they will need to be proficient in genomics in their future practice, to enable diagnoses^10^, patient management^16^ and access to targeted treatments or personalized medicine^17^. Referral to genetics can be a barrier to timely genomic medicine^18^, which can lead to delayed management of genetic disease. Non-genetics specialists also recognize the need for education in genomic medicine^10,19,20^. The need to provide genomics education to health professionals has been recognized around the world by many national genomics initiatives charged with progressing genomic medicine^1^. The goal of genomics education should meet both the needs of the target audience and the requirements of the intended service delivery model for genomic medicine^18^. For example, physicians’ education needs to identify patients who may benefit from genomic testing and refer appropriately to genetics services, are likely to differ from those in a service model where they also request tests themselves^7^. Most countries have not prescribed a single approach to implementing genomic medicine, but studies by our group and collaborators found 41^10^–77%^21^ of Australian physicians prefer a model of referring patients to genetics services.

Education can be tempered by the need for organizational as well as individual change^22^. A number of theories and approaches can be used to describe how innovative practice spreads throughout healthcare systems, focusing on the nature of the innovation itself^23^, the readiness of the system or environment for change, and the characteristics of the adopter themselves^22^. Several studies have suggested demographic and practice variables are associated with genetic/genomic testing practice^15,16,20,24,25^.

Training and education have also been associated with increased genomic confidence^16^ and genomic testing referral^15^. American physicians’ confidence in their genomic knowledge, communication and practice ability were associated with their intent to test, and likelihood to disclose, actionable genomic results^24^. In Australia, a qualitative study found involvement in research influenced physicians’ use of genomics in their clinical practice^25^ and dermatologists were more likely to discuss or offer testing if they perceived its relevance to current practice^16^.

To inform education as a strategy to optimize adoption of genomics by non-genetics medical specialists, we investigated physicians’ practices, perceptions and preferences relating to genomic testing and education through a national survey^10,17^. A behavior change theoretical framework, encompassing capability, opportunity and motivation (COM-B) was integral to its development^26^. The behavior was defined as engagement in genomic testing practices, with confidence to practice related to motivation to perform the behavior^27^. While 42% of respondents in our recent survey of Australian physicians stated that improved genomics knowledge may alter their clinical practice, the same proportion (42%) were ‘unsure’^10^. This suggested further exploration of the role of genomics education in preparing the non-genetics specialist workforce was warranted.

We therefore tested three hypotheses that

1. engagement with genomic medicine would be associated with respondent demographics (career stage, location, specialty, being a researcher or previously completing genomics education), perceptions of genomic medicine (confidence, preparedness, and/or preferences for models of practicing genomic medicine in the future);
2. confidence to practice genomic medicine would be associated with respondent demographics, current clinical genomics practice, perceptions of genomic medicine (proximity, preparedness, and/or service model preferences);
3. learning about particular genomics education topics would be associated with demographics, engagement, confidence and perceptions of genomic medicine.

## Results

**Fig. 1:**
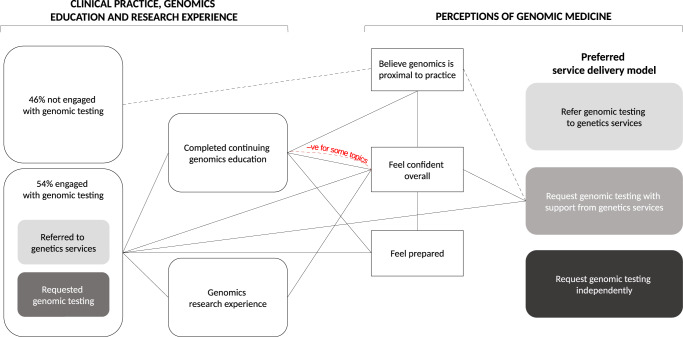
Results of association analyses. All connector lines present significant associations. Solid connector lines show observed positive associations. The dashed black connectors show that those who were not engaged but thought genomics was proximal preferred a model of request with support from genetics. The dashed red connector shows a negative association between confidence and having learnt about topics on genetics knowledge or clinical aspects.

The sample characteristics and cross-sectional results for the 409 physician respondents have been reported previously^10^. The results of association analyses relating to current engagement with genomic medicine (hypothesis 1), confidence to practice (hypothesis 2), and genomics education (hypothesis 3), and preferences for service models, are described below and summarized in Fig. 1.

### Engagement with genomic medicine

Those who were engaged in genomics (‘engaged respondents’) were more likely to be pediatricians, to have completed continuing genomics education (CGE) in the past year, and/or to have had genomics research experience (*p* < 0.001 for all; Table 1). There were no differences in engagement at different career stages; association analyses for practice location were underpowered.

**Table 1:** Associations between engagement with genomic medicine and demographics, current education and research experience and perceptions about genomic medicine.

Characteristic	Details	(total *n*) *n*	Not engaged (%; 99% CI)	Engaged (%, 99% CI)	*χ*^2^, *p*
*Demographics*					
Specialty		(386)			32.05, <0.001
	Pediatricians	76	13	63	
			(17.1; 7.7–30.8)	(82.9; 69.2–92.3)	
	Other specialties	310	165	145	
			(53.2; 45.9–60.6)	(46.8; 39.6–54.1)	
Career stage		(386)			0.10, 0.95
	Early (<10 years)	27	12	15	
			(44.4; 21.4–70.1)	(55.6; 29.9–78.6)	
	Mid (10–20 years)	110	52	58	
			(47.3; 35.2–59.7)	(52.7; 40.3–64.8)	
	Late (>20 years)	249	114	135	
			(45.8; 37.8–54.0)	(54.2; 47.0–62.2)	
Location	(underpowered)				–
*Current genomics education and research experience*					
Completed continuing genomics education in last year		(273)			30.92, <0.001
	Yes	92	17	75	
			(18.5; 9.4–31.0)	(81.5; 69.0–90.6)	
	No	181	97	84	
			(53.6; 43.9–63.0)	(46.4; 37.0–56.1)	
Genomics research experience		(386)			56.85, <0.001
	Yes	95	12	83	
			(12.6; 5.4–23.8)	(87.4; 76.2–94.6	
	No	291	166	125	
			(57.0; 49.4–64.4)	(43.0; 35.6–50.6)	
*Perceptions of genomic medicine*					
Overall feel confident to practice genomic medicine		(386)			30.57, <0.001
	Yes	229	79	150	
			(34.4, 27.2–47.5)	(65.5, 56.9–73.4)	
	No	157	99	58	
			(63.1, 52.6-72.4)	(36.9, 25.6-47.4)	
Feel prepared to practice genomic medicine		(301)			47.18, <0.001
	Yes	73	6	67	
			(8.2, 2.8–21.7)	(91.8, 78.3–97.2)	
	No	224	121	103	
			(54.0, 45.3–62.5)	(46.0, 37.5–54.7)	
Preference for service model of request genomic testing with support from genetics services rather than refer to genetics services	*Inpatients*	(178)			27.54, <0.001
	Yes	43	4	39	
			(9.3; 3.4–22.8)	(90.7; 77.2–96.6)	
	No	135	74	61	
			(54.8; 46.3–63.1)	(45.2; 48.3–78.2)	
	*Outpatients*	(195)			13.89, <0.001
	Yes	49	9	40	
			(18.4; 9.7–32.1)	(81.6; 67.9–90.3)	
	No	146	71	75	
			(48.6; 40.6–56.8)	(51.4; 43.2–49.4)	

Engaged respondents were more likely to feel confident (*p* < 0.001) and prepared (*p* < 0.001) to practice genomic medicine than those who were not engaged (Table 1). When asked about preferred service models for future practice, association analyses revealed engaged respondents were more likely to prefer to request genomic testing themselves with additional support from genetics services than other models, such as referring to genetics services, for both inpatients and outpatients (*p* < 0.001 for both; Table 1). Regression analyses revealed they were 10.63 times more likely to prefer this model with inpatients (99% CI 2.74–41.27, *p* = 0.004) and 4.21 times more likely with outpatients (99% CI 1.48–1.92, *p* < 0.001). After controlling for specialty, being engaged with genomics still predicted a preference for a model of ‘request with support from genetics services’ over any other model for both inpatients (OR 8.44, 99% CI 2.11–33.65, *p* < 0.001) and outpatients (OR 3.56, 99% CI 1.25–10.15, *p* = 0.002).

For respondents who were *not* engaged with genomics, the most common reason for not requesting or referring for a genomic test was being unsure about relevance to clinical practice (47% of respondents for panel tests; 50% for E/GS tests; Supplementary Table 1). Those who were not engaged but expected genomics to impact their practice in the next 2 years preferred a model of requesting genomic testing themselves with support from genetics services for inpatients (*p* = 0.007); there was no significant association for outpatients.

### Confidence to practice genomic medicine

More than half of respondents reported being confident in each aspect of knowledge about genomics and clinical skills in practicing genomic medicine (Table 2), with 42% being confident for all four aspects.

**Table 2:** Confidence in different aspects of knowledge and skills relating to genomic medicine (*N* = 273).

	% reported being confident	95% CI
Knowledge about genomics	57.7	52.8–62.5
Ability to elicit information about genetic conditions as part of a family or medical history	79.4	75.2–83.3
Ability to explain genomic concepts to patients	66.0	61.2–70.6
Ability to make decisions based on genomic information	61.1	56.2–65.9

Calculating average confidence across the four aspects, overall confidence was associated with being a pediatrician, already being engaged with genomics, having completed CGE and having research experience (*p* < 0.001 for all; Table 3). There was no association between overall confidence and career stage or practice location. Those who were confident were more likely to have genomics research experience (*p* < 0.001), believe genomics will impact their practice in the next 2 years (*p* < 0.001) and to feel prepared to practice genomic medicine (*p* < 0.001) than those who were not confident. Those who were confident also preferred a service model of requesting genomic testing themselves with support from genetics services for both inpatients and outpatients (*p* = 0.002 and *p* = 0.001, respectively; Table 3). Regression analyses revealed those who were confident were 3.05 times more likely to prefer this model with inpatients (99% CI 1.20–7.79, *p* = 0.002) and 3.21 times more likely with outpatients (99% CI 1.32–7.81, *p* = 0.001). After controlling for specialty, being confident still predicted a preference for a model of ‘request with support from genetics services’ over any other model for outpatients (OR 2.81, 99% CI 1.11–7.08, *p* = 0.004) but only approached significance for inpatients (*p* = 0.031).

**Table 3:** Associations between overall confidence to practice genomic medicine and demographics, current practice and opinions about genomic medicine.

Characteristic	Details	(total *n*) *n*	Not confident (%, 99% CI)	Confident (%, 99% CI)	*χ*^2^, *p*
*Demographics*					
Specialty		(409)			12.93, <0.001
	Pediatricians	81	140	188	
			(42.7; 35.8–49.9)	(57.3; 50.1–64.2)	
	Other specialties	328	17	64	
			(21.0; 11.4–35.3)	(79.0; 64.7–88.6)	
Career stage		(409)			1.73, 0.42
	Early (<10 years)	27	13	14	
			(48.2; 24.2–73.0)	(51.8; 27.0–75.8)	
	Mid (10–20 years)	116	47	69	
			(40.5; 29.3–52.8)	(59.5; 47.2; 70.7)	
	Late (>20 years)	266	97	169	
			(36.5; 29.2–44.4)	(63.5; 55.6–70.8)	
Location		(408)			0.99, 0.61
	Metropolitan	306	114	192	
			(37.2; 30.4–44.7)	(62.8; 55.3–69.6)	
	Inner regional	59	26	33	
			(44.1; 28.2–61.3)	(55.9; 38.7–71.9)	
	Outer regional, remote, very remote	43	17	26	
			(39.5; 22.0–60.3)	(60.5; 39.7–78.0)	
*Clinical practice, genomics education and research experience*					
Currently engaged with genomics		(386)			30.6, <0.001
	Yes	208	58	150	
			27.9 (20.6–36.6)	(72.1, 63.4–79.4)	
	No	178	99	79	
			55.6 (45.8–65.0)	(44.4, 35.0–54.2)	
Completed continuing genomics education in last year		(273)			56.07, <0.001
	Yes	92	24	68	
			(26.1; 15.9–39.7)	(73.9; 60.3–84.1)	
	No	181	133	48	
			(73.5;64.1–81.1)	(26.5; 18.9–35.9)	
Genomics research experience		(388)			22.63, <0.001
	Yes	96	19	77	
			(19.8; 11.2–32.6)	(80.2; 67.4–88.8)	
	No	292	138	154	
			(47.3; 39.8–54.8)	(52.7; 45.2–60.2)	
*Perceptions of genomic medicine*					
Genomic medicine will impact clinical practice within 2 years (proximity)		(298)			47.0, <0.001
	Yes	199	77	122	
			(38.7, 30.2–48.0)	(61.3, 52.0–69.8)	
	No	99	80	19	
			(80.8, 68.3–89.2)	(19.2, 10.8–31.7)	
Feel prepared to practice genomic medicine		(297)			68.2, <0.001
	Yes	224	8	65	
			(11.0, 4.4–24.9)	(89.0, 75.1–95.6)	
	No	73	148	75	
			(66.5, 57.9–74.2)	(33.5, 25.8–42.1)	
Preference for service model of request genomic testing with support from genetics services rather than refer to genetics services	*Inpatients*	(178)			9.92, 0.002
	Yes	43	16	27	
			(37.2; 20.2–58.1)	(62.8; 41.9–79.8)	
	No	135	87	48	
			(64.4; 53.1–74.4)	(35.6; 35.6–46.9)	
	*Outpatients*	(195)			11.94, 0.001
	Yes	49	17	32	
			(34.7; 19.2–54.3)	(65.3; 45.7–80.8)	
	No	146	92	54	
			(63.0; 52.1–72.7)	(36.9; 27.3–47.9)	

### Learning about particular genomics education topics

Respondents who stated improved genomics knowledge would alter their practice were more likely to have completed CGE in the past year (85.5% vs 63.9%, *p* = 0.003). Of respondents who had completed CGE, those who were engaged with genomics reported higher average confidence than those who were not engaged (*p* < 0.001).

At least half the respondents had previously learnt topics within the categories of genetics/genomics knowledge (mean 53.3%, range 55.7–77.5%) and clinical aspects (53.3%, 52.0–70.8%), with fewer learning about technology (46.9%, 37.6–64.9%) and ethical, legal and social implications (ELSI) (41.1%, 52.4–59.0%) topics.

Supplementary Table 2 summarizes associations between each topic category and variables related to demographics, current practice and perceptions of genomic medicine. Pediatricians were more likely to have learnt each topic category than other specialties (*p* < 0.001 for all); there were no associations between either career stage or location. Those already engaged with genomics were also more likely to have learnt about each topic category (*p* < 0.001 for all). Those who thought genomics was proximal or who felt prepared were more likely to have learnt about each topic category (*p* < 0.001 for all). Those who were confident were *less* likely to have learnt about topics related to genetics/genomics knowledge or clinical pre- or post-test aspects (*p* < 0.001 for both); there were no associations between confidence and topics relating to learning about testing technology or ELSI. There were no associations between service model preferences and having learnt about any topic category.

The topic most commonly learnt about by respondents who were already engaged with genomics, or who felt confident, was ‘basic concepts’ in genetics/genomics knowledge (Supplementary Table 3). After controlling regression analyses for specialty, having previously learnt about any topic category predicted engagement with genomics, perceptions of proximity to clinical practice, and feeling prepared (*p* < 0.001 for all; Table 4). However, those who had learnt genetics/genomics knowledge, clinical pre- or post-test aspects or testing technologies were 1.8–3.0 times *less* likely to be confident (*p* < 0.001, *p* < 0.001 and *p* = 0.01, respectively). There was no evidence of a difference in overall confidence for those who had learnt about ELSI topics, and no evidence for differences in service model preferences. Regression analyses that were not controlled for specialty showed similar results.

**Table 4:** Regression analyses predicting engagement, confidence, proximity, preparedness and preferred service model from topics learnt, controlled for specialty.

Variable	Genetics/genomics knowledge	Clinical aspects	Testing technology	ELSI
	OR (99% CI, *p*)			
*Current genomics education and practice*				
Engaged with genomics (requested or referred for a panel or E/GS in the past year)	2.87	3.66	3.02	3.69
	(1.62–5.10, <0.001)	(2.04–6.57, <0.001)	(1.70–5.36, <0.001)	(2.03–6.69, <0.001)
*Perceptions of genomic medicine*				
Genomic medicine will impact clinical practice within 2 years (proximity)	3.34^a^	3.21^a^	2.86^a^	4.52
	(1.62–6.89, <0.001)	(1.61–6.43, <0.001)	(1.39–5.86, 0.001)	(2.23–9.16, <0.001)
Feel prepared to practice genomic medicine	4.59^a^	3.40^a^	4.59^a^	7.49
	(1.47–14.29, <0.001)	(1.14–10.15, 0.004)	(1.57–14.29, 0.001)	(2.82–19.93, <0.001)
Overall feel confident to practice genomic medicine	0.34	0.33	0.57	1.10
	(0.19–0.61, <0.001)	(0.18–0.58, <0.001)	(0.33–1.00, 0.010)	(0.63–1.92, 0.665)
Preference for service model of ‘request testing with support from genetics services’^b^				
Inpatients	0.43^a^	0.41^a^	0.41^a^	0.66^a^
	(0.08–2.33, 0.198)	(0.10–1.74, 0.192)	(0.10–1.74, 0.111)	(0.21–2.03, 0.336)
Outpatients	0.64^a^	0.63^a^	0.63^a^	0.52^a^
	(0.17–2.34, 0.374)	(0.20–1.99, 0.247)	(0.20–1.99, 0.300)	(0.19–1.48, 0.108)

Supplementary Table 1 lists topics within each category.

^a^A Firth logistic regression was conducted due to insufficient cell frequencies.

^b^Due to the very low frequency of respondents who indicated they preferred to initiate testing themselves in the future, that category was removed from these analyses.

Respondents were at least twice as likely to be engaged if they indicated a preference for learning topics in the future relating to genetics/genomics knowledge (*p* = 0.002), clinical aspects (*p* < 0.001) or testing technology (*p* < 0.001; Supplementary Table 4); a desire to learn about ELSI topics in the future did not quite predict engagement (*p* = 0.011). Respondents were 6.7–16.7 times *less* likely to currently feel confident if they indicated a preference for learning about any topic category in the future (*p* < 0.001 for all; Supplementary Table 4). Very few respondents (11/271; 4.1%) suggested an additional topic they would like to learn about beyond those listed; the few suggestions included pharmacogenetics, cost-benefit analyses of genomic testing and specialty-specific concepts, e.g., for geriatrics or anesthetics.

## Discussion

Understanding the characteristics of those already engaged with genomics and those who feel confident to practice can inform learning needs and help educators provide genomics education that is fit for purpose. Recognizing the relationships between engagement, confidence to practice genomic medicine, education, and preferred service models may also help define the role of education within broader implementation strategies. Figure 1 summarizes the relationships identified in our empirical data.

In our sample of Australian physicians not trained in genetics, those already engaged with genomics and those who felt confident were both more likely to be pediatricians with recent genomics education experience. The association with pediatrics may reflect the recognized relevance of genomics to pediatric practice^28^ and/or the close relationship between the fields of clinical genetics and pediatrics in identifying the genetic causes of childhood conditions^29^. In Australia, clinical genetics services are often co-located with pediatric hospitals, providing greater accessibility; as the majority of our sample were pediatricians, this may explain our finding that respondents were 2.5 times more likely to prefer a model of requesting testing with support from genetics services for inpatients than for outpatients. When examining respondents’ education experiences more closely, there were no associations between having learnt particular topic categories and career stage or location; this suggests that educators can develop programs for broad audiences, without the need to customize content.

Our results suggest that the practice of genomics education should be considered an ongoing or iterative process rather than a single interaction that enables genomic medicine. Prior learning of all four topic categories predicted being engaged with genomics. However, prior learning of topics relating to knowledge or clinical aspects predicted a *drop* in confidence. Respondents who had completed any form of CGE *and* were engaged with genomics were more confident than those who had completed CGE but were *not* engaged, suggesting that education alone is not sufficient to gain confidence in implementing genomic medicine in clinical practice^30^. It may even be that completing CGE causes a physician to move from ‘unconscious incompetence’ to ‘conscious incompetence’^31^. Certainly, short-format CGE is not currently designed for, or sufficient to, help physicians move along the continuum toward ‘conscious competence’ and ultimately ‘unconscious competence’. Therefore, perhaps the goal (and evaluation) of CGE for physicians should not be to enable sufficient confidence so that physicians can request genomic tests without support from genetics services but instead to provide a foundation for initial, facilitated engagement with genomics. Practice-based learning supplementary to CGE would then aim to increase confidence over time. As implementation of genomic medicine progresses, opportunities for practicing physicians to become immersed in workplace environments that provide opportunities to gain and apply new genomic knowledge and skills will increase. This will include learning from peers^22,25^ and supervised workplace learning^32^ to implement genomics in routine clinical practice.

**Fig. 2:**
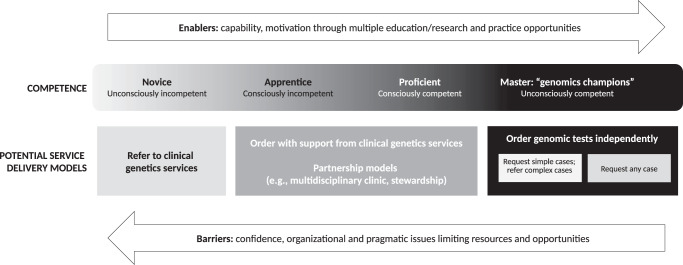
A proposed alignment between genomics competence and potential service delivery models.

Based on our findings, we propose Fig. 2, where a progression of genomic competence is aligned with a series of potential service delivery models, and education is one enabler of mastery or independence in ordering genomic tests. For example, once a (unconsciously incompetent) learner is aware of the potential clinical utility (relative advantage) of genomics and its value for their practice (compatibility)^23^, they may undertake education that covers genetics/genomics knowledge, clinical aspects of testing, testing technologies and the associated ethical, legal and social issues. At that stage, a non-genetics physician may realize the complexity of the testing and results, reducing their confidence (conscious incompetence). They may then prefer a service model that involves referral to a genetics service and counselling. The opportunity to practice genomic medicine in a clinical or research setting (trial) or observe genomics through peer-to-peer learning or multidisciplinary teams (observability) could provide additional impetus for adoption^33^, and a potential shift in preference for service delivery to ordering testing with support from genetics, or similar “partnership” models^7,18^. Recent Australian education programs offered as part of translational research studies for acute care and renal genomics, have been designed to include opportunities for multidisciplinary engagement (see www.eventbrite.com.au/e/rapid-genomics-in-the-nicupicu-tickets-126912585961 and www.eventbrite.com.au/e/clinical-genomics-for-kidney-disease-tickets-221737010367). Engagement may highlight the speed of change, prompting further physician interest in mastering and maintaining their knowledge, or create the opportunity to shift roles from a type of apprenticeship to more independent practice^33^.

Our results also highlight the need to provide a range of education formats and topics pitched to different levels of competence along the proposed progression, independent of career stage^34^. This would allow physicians to upskill and refresh their knowledge to complement their experience. It also suggests that educational opportunities to upskill for a new independence may also be more effective at particular stages of a physician’s progression. For example, a physician who has been referring to genetics and observing multidisciplinary team discussions of patient intake and result interpretation may realize that they need upskilling in the particular pathways and procedures for ordering testing, or a physician who is already requesting genomic tests may recognize that upskilling in patient communication and counselling skills is an important step toward independent practice.

Our survey was deployed in 2019, before Australian government-funded exome/genome sequencing (E/GS) testing became available for certain conditions^35^. At that time, the majority of Australian physicians preferred a future service model where they continued to refer patients requiring genomic testing to a clinical genetics service, for both inpatients and outpatients^10^. However, we found that physicians who were engaged or confident were more likely to prefer a service model of requesting genomic testing themselves with support from genetics services. With the advent of funded tests, more physicians may become engaged and their service model preferences may change—longitudinal studies would be beneficial. The challenge for clinical genetics services will be to anticipate and respond to this evolution when determining workforce and service delivery. Increased genomic testing by non-genetics specialists increases the demand for genetics services, through both referral and seeking advice^7^. Unfortunately, the provision of advice to doctors is not currently funded in the Australian health system, amplifying the challenges for clinical genetics services.

Our findings represent perspectives on genomic medicine practice and education from the broadest range of medical subspecialties reported in the literature to date^10^. However, despite extensive efforts to disseminate the survey, our data represent only 3% of Australian physicians^10^, and the sample size limited the power of some statistical analyses. Many survey measures were subjective and all were self-reported. Those who completed the survey may have had an interest in genomic medicine, introducing some responder bias, but they are also therefore likely to be potential consumers of CGE, providing valuable insights to guide educators. Questions around prior learning of genomics did not specify timelines, so some respondents may have reflected on medical school teaching, which may have been many years prior.

‘Engagement’ was defined to reflect physician behavior related to requesting or referring for genomic tests, with consideration that engagement with genomics differs by medical specialty, role and external factors, such as test access and funding. In some practices, very small numbers per year might be appropriate as requesting or referring for genomic testing is dependent on the patients seen during that period. Questions about requesting or referring to genetics services for genomic testing were combined in our survey, which limited insights into whether physicians engage with both behaviors. Future modifications of the survey could make these questions independent. Our aim is that this survey, our empirical evidence, and our proposed model of a progression of genomic competency aligned with potential service delivery models, will serve as a basis to understand physician behavior and inform others in defining what constitutes a ‘sufficient’ level of engagement in their local context.

Most respondents indicated they would like to learn more about the topics they had already learnt about, which suggests appropriateness, availability and accessibility of current genomics education offerings. We cannot, however, comment on depth of knowledge required across the topics to progress to the different stages of competency shown in Fig. 2.

Our findings support a medical education model that focuses on outcomes and can be tailored to individualized learning pathways. The model should also acknowledge the value of genomics engagement and experience in both formal and informal settings, and the influence of implementation strategies and service delivery models on education goals and outcomes.

## Methods

### Context

In Australia, genomic testing is still predominantly offered through clinical genetics services or in a research setting. Clinical genetics services are primarily based in public hospitals; specialist health professionals (e.g., clinical/medical geneticists and genetic counsellors) provide screening, diagnostic testing, and genetic counselling on referral. Patients receive some reimbursement for private consultations and specified pathology tests through the federally-funded Medicare Benefits Scheme (MBS). At the time of our study, tests for only 20 genetic conditions were reimbursed through the MBS^10^. Since then, reimbursement for genomic tests has been included for certain pediatric, cardiology or nephrology conditions with some still requiring review by clinical genetics services to be eligible for MBS funding.

Recognizing that the term ‘physician’ has different meanings in different countries, here we define ‘physicians’ as doctors who have completed Basic Training and either commenced or completed Advanced Training as medical specialists and passed examinations to obtain fellowship of the relevant medical college and/or professional society (e.g., the Royal Australasian College of Physicians). For the purposes of this Australian study, we exclude general practitioners (‘family physicians’) from this definition. Here, we focus on the non-genetics medical workforce, i.e., physicians who are trained or in training for a specialty other than clinical genetics. We define ‘clinical genomic testing’ as testing that investigates many regions of the genome at once to investigate genetic conditions, such as gene panels and E/GS, but excluding non-invasive prenatal testing using sequencing technologies.

### Survey

Details of survey development, design and deployment have been reported elsewhere^10,17^. Briefly, the survey included five domains that were identified through exploratory qualitative interviews^25^, with 28 questions on respondent demographics and employment, current and expected clinical genomics practice, perceptions of proximity and preparedness to practice, and experience and preferences for CGE. Questions about current practice focused on respondents’ behavior related to requesting or referring for genomic testing in the past year. Confidence in genomics was determined using a previously-validated scale that assesses confidence across four aspects of knowledge and clinical skills, including identifying patients who could benefit from testing, genomics communication and decision making^24^. Options for future service delivery model preferences for both inpatients and outpatients were that respondents: request genomic testing themselves; request themselves with support from genetics services (such as advice on appropriate test type, interpreting results or communicating with patients and families); refer to genetics services; or unsure. Respondents were asked if they had attended any CGE in the past 2 years, and which topics related to genomics they had learnt about or would like to learn about (topics listed in Supplementary Table 5).

The survey was advertised via medical college and society membership newsletters and mailings, hospital staff lists, social media, and investigator and respondent networks. Inclusion criteria were medical doctors who had commenced or completed their specialist training and were currently practicing clinically in Australia (‘physicians’). Five specialties were excluded because they were either the focus of separate studies (medical geneticists^36^, oncologists (ongoing), family physicians^37^), or because they do not request genomic tests in the Australian health system (radiologists and pathologists).

Participants completed the survey online from February–September 2019, providing consent by completing a screening and consent question at commencement. Data were collected using Research Electronic Data Capture software hosted at the Murdoch Children’s Research Institute^38^. Ethics approval was provided by The University of Melbourne, Melbourne, Australia (HREC #1646785).

### Data analysis

Data were exported, cleaned to remove incomplete responses (no data beyond demographic questions) then analyzed in Stata 16.0 (Stata Corporation, College Station, TX, USA). ‘Career stage’ was defined as early (<10 years since medical degree completed), mid (10–20 years) or senior (>20 years). ‘Engagement with genomic medicine’ was defined as requesting or referring for a panel or E/GS test in the past year. ‘Overall confidence’ was calculated as an average across all four aspects, converted to a binary variable (1–5 = not confident; 6–10 = confident), to be consistent with measures for other variables and to achieve sufficient power.

Learning topics in genomic medicine (21 in total) were grouped into four categories: ‘genetics/genomics knowledge’, genetic/genomic testing technologies (‘technology’); clinical pre- or post-test aspects (‘clinical aspects’); and ‘ELSI’ (Supplementary Table 5). Respondents were classified as learning about a topic category if they selected at least one item within a category, as the majority indicated having previously learnt either none or all possible topics within each category (Supplementary Fig. 1).

Descriptive and inferential analyses included two-sample tests of proportions, chi-square, Fisher’s exact tests, Kruskal–Wallis tests, or independent-sample *t*-tests. Bivariate logistic regressions were used for most regression analyses with Firth logistic regressions used to reduce bias when frequency distributions resulted in small expected cell sizes (>20% had <5 expected cases). The outcome variables for the regression analyses were engagement (hypothesis 1), confidence (hypothesis 2) and genomics education topic categories (hypothesis 3). Where there was an association between specialty and a variable, regression analyses were adjusted for specialty, as pediatricians were the largest group of respondents (20%) and more likely to be engaged with genomics than other specialties. The small numbers of respondents who preferred a service delivery model of requesting testing themselves without support from genetics services, or who were unsure, were excluded from regression analyses due to small sample sizes. A *p* value of <0.01 was considered significant.

### Reporting summary

Further information on research design is available in the Nature Research Reporting Summary linked to this article.

## Supplementary information

Reporting Summary

Supplementary Information

## Data Availability

The dataset generated during and/or analyzed during the current study are available from the corresponding author on reasonable request.
