# Supplementary material for: Investigating genomic medicine practice and perceptions amongst Australian non-genetics physicians to inform education and implementation
Source: NPJ Genom Med. 2023 Jun 24;8:13. doi: 10.1038/s41525-023-00360-1 (PMC10290687; doi:10.1038/s41525-023-00360-1)
Supplement: Supplementary file 1 — Reporting Summary [file 41525_2023_360_MOESM1_ESM.pdf]

nature portfolio

Corresponding author(s): Amy Nisselle

Last updated by author(s): 2023/05/08

Reporting Summary

Nature Portfolio wishes to improve the reproducibility of the work that we publish. This form provides structure for consistency and transparency in reporting. For further information on Nature Portfolio policies, see our Editorial Policies and the Editorial Policy Checklist.

Statistics

For all statistical analyses, confirm that the following items are present in the figure legend, table legend, main text, or Methods section.

n/a	Confirmed	
☐	☒	The exact sample size (n) for each experimental group/condition, given as a discrete number and unit of measurement
☐	☒	A statement on whether measurements were taken from distinct samples or whether the same sample was measured repeatedly
☐	☒	The statistical test(s) used AND whether they are one- or two-sided<br>*Only common tests should be described solely by name; describe more complex techniques in the Methods section.*
☐	☒	A description of all covariates tested
☐	☒	A description of any assumptions or corrections, such as tests of normality and adjustment for multiple comparisons
☐	☒	A full description of the statistical parameters including central tendency (e.g. means) or other basic estimates (e.g. regression coefficient) AND variation (e.g. standard deviation) or associated estimates of uncertainty (e.g. confidence intervals)
☒	☐	For null hypothesis testing, the test statistic (e.g. F, t, r) with confidence intervals, effect sizes, degrees of freedom and P value noted<br>*Give P values as exact values whenever suitable.*
☒	☐	For Bayesian analysis, information on the choice of priors and Markov chain Monte Carlo settings
☒	☐	For hierarchical and complex designs, identification of the appropriate level for tests and full reporting of outcomes
☒	☐	Estimates of effect sizes (e.g. Cohen's d, Pearson's r), indicating how they were calculated

Our web collection on statistics for biologists contains articles on many of the points above.

Software and code

Policy information about availability of computer code

Data collection	N/A
Data analysis	N/A

For manuscripts utilizing custom algorithms or software that are central to the research but not yet described in published literature, software must be made available to editors and reviewers. We strongly encourage code deposition in a community repository (e.g. GitHub). See the Nature Portfolio guidelines for submitting code & software for further information.

Data

Policy information about availability of data

All manuscripts must include a data availability statement. This statement should provide the following information, where applicable:

- Accession codes, unique identifiers, or web links for publicly available datasets
- A description of any restrictions on data availability
- For clinical datasets or third party data, please ensure that the statement adheres to our policy

The dataset generated during and/or analysed during the current study are available from the corresponding author on reasonable request.

Research involving human participants, their data, or biological material

Policy information about studies with human participants or human data. See also policy information about sex, gender (identity/presentation), and sexual orientation and race, ethnicity and racism.

Reporting on sex and gender	N/A
Reporting on race, ethnicity, or other socially relevant groupings	N/A
Population characteristics	Demographics: Medical specialty, career stage, location<br>‘Engagement with genomic medicine’<br>‘Confidence in genomics'<br>'Learning topics in genomic medicine', 21 in total, grouped into four categories: ‘genetics/genomics knowledge’, genetic/genomic testing technologies (‘technology’); clinical pre- or post-test aspects (‘clinical aspects’); and ethical, legal, and social implications of genetic/genomic testing (‘ELSI’).
Recruitment	Described in previous publication (ref #10 in manuscript)
Ethics oversight	The University of Melbourne, Australia

Note that full information on the approval of the study protocol must also be provided in the manuscript.

Field-specific reporting

Please select the one below that is the best fit for your research. If you are not sure, read the appropriate sections before making your selection.

☐ Life sciences ☒ Behavioural & social sciences ☐ Ecological, evolutionary & environmental sciences

For a reference copy of the document with all sections, see nature.com/documents/nr-reporting-summary-flat.pdf

Behavioural & social sciences study design

All studies must disclose on these points even when the disclosure is negative.

Study description	Quantitative cross-sectional
Research sample	Australian physicians not trained in genetics, oncology or general practice ('family medicine')
Sampling strategy	Described in previous publication (ref #10 in manuscript). Briefly, attempts were made to reach all Australian physicians not trained in genetics, oncology or family medicine, through professional organisations and societies, social media and snowball sampling
Data collection	Described in previous publication (ref #10 in manuscript), cheifly anonymous online survey hosted in REDCap
Timing	Described in previous publication (ref #10 in manuscript), March-October 2019
Data exclusions	Described in previous publication (ref #10 in manuscript), no data were excluded
Non-participation	Described in previous publication (ref #10 in manuscript), RR ~3% of eligible Australian physicians
Randomization	N/A

Reporting for specific materials, systems and methods

We require information from authors about some types of materials, experimental systems and methods used in many studies. Here, indicate whether each material, system or method listed is relevant to your study. If you are not sure if a list item applies to your research, read the appropriate section before selecting a response.

Materials & experimental systems

n/a	Involved in the study
☒	☐ Antibodies
☒	☐ Eukaryotic cell lines
☒	☐ Palaeontology and archaeology
☒	☐ Animals and other organisms
☒	☐ Clinical data
☒	☐ Dual use research of concern
☒	☐ Plants

Methods

n/a	Involved in the study
☒	☐ ChIP-seq
☒	☐ Flow cytometry
☒	☐ MRI-based neuroimaging
