# Supplementary material for: Investigating genomic medicine practice and perceptions amongst Australian non-genetics physicians to inform education and implementation
Source: NPJ Genom Med. 2023 Jun 24;8:13. doi: 10.1038/s41525-023-00360-1 (PMC10290687; doi:10.1038/s41525-023-00360-1)
Supplement: Supplementary file 2 — Supplementary Information [file 41525_2023_360_MOESM2_ESM.docx]

# Supplementary Information

# Nisselle et al. Investigating genomic medicine practice and perceptions amongst Australian non-genetics physicians to inform education and implementation.

#### Supplementary Table 1: Reasons for not being engaged with genomic medicine in the past year.*

| **Reason** | **Panel tests** | | **Exome/genome sequencing tests** | |
| --- | --- | --- | --- | --- |
|  | **N=191**^1^ | | **N=233**^1^ | |
|  | **n** | **%** | **n** | **%** |
| I am unable to order a [gene panel/ E/GS] test in my current role/ department, e.g., lack of access to testing or funding | 5 | 3 | 7 | 3 |
| I’m not sure how to order a [gene panel/ E/GS] test | 34 | 18 | 33 | 14 |
| I’m not sure of the relevance of [gene panel/ E/GS] to my practice | 90 | 47 | 117 | 50 |
| It’s not my responsibility | 2 | 1 | 1 | 0 |
| Other (“not in routine practice”) | 1 | 1 | 3 | 1 |

^1^ Engagement was defined as requesting a genomic test or referring patients to clinical genetics services. N is the number of respondents who indicated they neither requested or referred for a test.

#### Supplementary Table 2: Associations between having learnt each category of genomics education with demographics, current practice and opinions about genomic medicine.^1^

| **Variable** | **Response grouping** | | **(total N)**  **n** | **Genetic/genomic knowledge** (4 topics) | | **Clinical aspects** (7 topics) | | **Testing technology** (7 topics) | | **ELSI** (3 topics) |  |
| --- | --- | --- | --- | --- | --- | --- | --- | --- | --- | --- | --- |
|  |  |  |  | χ**^2^**, *p* % (99%CI) for each variable response option | | | | | | |  |
| **Demographics** | | |  |  | |  | |  | |  |  |
| Specialty | Pediatrics  Other specialties | | (409)  81  328 | 19.65, *<0.001*  75.3 (60.7–85.8)  47.9 (40.8–55.0) | | 19.65, *<0.001*  75.3 (60.7–85.8)  47.9 (40.8–55.0) | | 29.85, *<0.001*  74.1 (59.7–84.6)  40.2 (33.4–47.4) | | 27.33, *<0.001*  66.7 (52.1–78.6)  34.6 (28.3–41.8) |  |
| Career stage | Early (<10 years)  Mid (10–20 years)  Late (>20 years) | | (409)  27  116  266 | 3.15, *0.21*  40.7 (18.8–67.1)  58.6 (46.4–69.9)  52.2 (44.3–60.1) | | 1.01, *0.60*  44.4 (21.4–70.1)  55.2 (43.0–66.7)  53.4 (45.4–61.2) | | 0.49, *0.78*  40.7 (20.0–65.5)  46.6 (35.0–58.5)  47.7 (39.9–55.7) | | 0.72, *0.70*  33.3 (14.8–60.0)  41.4 (30.2–53.5)  41.7 (34.2–49.7) |  |
| Location | Metropolitan Inner regional  Outer regional/ remote/ very remote | | (408)  306  59  43 | 0.33, *0.85*  54.2 (46.8–61.5)  50.8 (34.1–67.4)  51.2 (31.5–70.5) | | 3.48, *0.18*  54.9 (47.5–62.1)  47.5 (31.5–63.9)  51.2 (31.5–70.5) | | 1.20, *0.55*  49.7 (42.3–57.0)  40.7 (25.7–57.7)  37.2 (20.8–57.3) | | 0.54, *0.76*  42.2 (35.1–49.6)  37.3 (22.9–54.4)  39.6 (22.6–59.4) |  |
| **Current practice** | |  | | |  | |  | |  | | |
| Engaged with genomics (requested or referred for a panel or E/GS in the past year) | Yes  No | | (386)  208  178 | 34.5, *<0.001*  70.2 (61.4–77.7)  40.4 (31.4–50.2) | | 37.0, *<0.001*  70.7 (61.9–78.1)  39.9 (30.9–49.7) | | 49.7, *<0.001*  66.3 (57.4–74.2)  30.3 (22.2–39.9) | | 47.5, *<0.001*  59.6 (50.6–68.0)  24.7 (17.3–34.0) |  |
| **Perceptions** | | |  |  | |  | |  | |  |  |
| Genomic medicine will impact clinical practice within two years (proximity) | Yes  No | | (298)  99  199 | 29.1, *<0.001*  82.9 (74.9–88.8)  53.5 (40.6–66.0) | | 23.4, *<0.001*  81.9 (73.7–87.9)  55.6 (42.6–67.9) | | 31.3, *<0.001*  75.4 (66.6–82.4)  42.4 (30.3–55.5) | | 44.2, *<0.001*  69.8 (60.8–77.6)  29.3 (18.9–42.3) |  |
| Feel prepared to practice genomic medicine | Yes  No | | (297)  73  224 | 16.7, *<0.001*  91.8 (78.7–97.2)  67.4 (58.8–75.0) | | 16.7, *<0.001*  91.8 (78.7–97.2)  67.4 (58.8–75.0) | | 29.1, *<0.001*  91.8 (78.3–97.2)  55.8 (47.1–64.2) | | 29.1, *<0.001*  87.7 (73.5–94.8)  46.4 (38.0–55.1) |  |
| Overall feel confident to practice genomic medicine | Yes  No | | (409)  252  157 | 15.5, *<0.001*  45.6 (37.7–53.8)  65.6 (55.2–74.7) | | 17.1, *<0.001*  45.2 (37.3–53.4)  66.2 (55.8–75.3) | | 2.2, *0.14*  44.0 (36.2–52.3)  51.6 (41.3–617) | | 1.8, *0.18*  43.7 (35.8–51.9)  36.9 (27.6–47.4) |  |
| Preference for service model (‘request testing with support from genetics services’ or ‘refer to genetics services)^2^ | Inpatients  Support  Refer  Outpatients  Support  Refer | | (111)  43  68  (136)  49  87 | 3.8, *0.06*  93.0 (72.6–98.5)  79.4 (63.5–89.5)  1.5, *0.22*  87.8 (69.7–95.7)  79.3 (65.7–88.4) | | 2.5, *0.19*  90.7 (70.3–97.6)  79.4 (63.5–89.5)  4.7, *0.04*  91.8 (74.2–97.8)  77.0 (63.3–86.7) | | 5.4, *0.02*  88.4 (67.8–97.5)  69.1 (52.7–81.8)  2.2, *0.14*  81.6 (62.9–92.1)  70.1 (56.0–81.2) | | 2.3, *0.13*  74.4 (53.1–88.2)  60.3 (44.0–74.6)  3.9, *0.05*  75.5 (56.4–88.0)  58.6 (44.5–71.4) |  |

^1^ **Supplementary Table 5** lists topics within each category.
^2^ Due to the very low frequency of respondents who indicated they preferred to initiate testing themselves in the future, this category was removed from these analyses.

#### Supplementary Table 3: Topics in genomics education that most respondents had learnt about, for those who were engaged with genomics (requesting or referring for a panel or E/GS test) or felt confident to practice genomic medicine^1^

| **Engaged** | | | **Confident** | | |
| --- | --- | --- | --- | --- | --- |
| **% of engaged** | **Category** | **Topic** | **% of confident** | **Category** | **Topic** |
| 69.7 | Genetics/ genomics knowledge | Basic concepts | 45.6 | Genetics/ genomics knowledge | Basic concepts |
| 64.9 | Genetics/ genomics knowledge | Disorders and diseases | 43.7 | Clinical pre- or post-test aspects | Recognising patients who may benefit from genomic testing |
| 64.4 | Testing technology | Different types of genetic tests | 43.3 | Genetics/ genomics knowledge | Disorders and diseases |
| 63.9 | Clinical pre- or post-test aspects | Recognising patients who may benefit from genomic testing | 43.3 | Testing technology | Different types of genetic tests |
| 63.9 | Clinical pre- or post-test aspects | Communication skills with patients | 42.1 | ELSI | Ethical implications |

^1^ **Supplementary Table 5** lists topics within each category.

#### Supplementary Table 4: Regression analyses predicting engagement and overall confidence from preferences for learning about genomics education topics^1^ in the future, controlled for specialty

| **Variable** | **Genetics/ genomics knowledge** | **Clinical aspects** | **Testing technology** | **ELSI** |
| --- | --- | --- | --- | --- |
|  | OR (99%CI, *p*) | | | |
| **Current genomics education and practice** | | | | |
| Engaged with genomics (requested or referred for a panel or E/GS in the past year) | 2.04 (1.14–3.66; *0.002*) | 2.01 (1.12–3.62; *<0.001*) | 2.30 (1.28–4.11; *<0.001*) | 1.73 (0.99–3.04; *0.011*) |
| **Perceptions of genomic medicine** | | | | |
| Overall feel confident to practice genomic medicine | 0.07 (0.02–0.14; *<0.001*) | 0.06 (0.03–0.13; *<0.001*) | 0.09  (0.04–0.18; *<0.001*) | 0.15  (0.08–0.28; *<0.001*) |

^1^ **Supplementary Table 5** lists topics within each category.

#### Supplementary Table 5. Topics in continuing genomics education

| **Genetics/genomics knowledge** | |
| --- | --- |
| 1. Basic concepts |  |
| 1. Disorders and diseases |  |
| 1. Current applications in genomic medicine |  |
| 1. Emerging applications in genomic medicine |  |
| **Clinical pre- or post-test aspects** |  |
| 1. Recognising patients who may benefit from genomic testing |  |
| 1. Communication skills with patients |  |
| 1. Performing genetic risk assessments i.e., family health history risk assessments |  |
| 1. Referring appropriately for a genomic test |  |
| 1. Requesting a genomic test for a patient, e.g., pathology form, blood tubes, consent |  |
| 1. Interpreting genomic test results |  |
| 1. Identifying additional family members who may benefit from genomic testing (cascade testing) |  |
| **Genetic/ genomic testing and technologies** |  |
| 1. Different types of genetic tests, e.g., microarray, single gene tests |  |
| 1. Different types of genomic tests, e.g., panels, whole exome/genome sequencing |  |
| 1. Different applications of somatic genomic tests |  |
| 1. Different applications of germline genomic tests |  |
| 1. Clinical utility of tests, e.g., diagnosis/ prognosis/ treatment/ ongoing management, including pharmacogenomics |  |
| 1. Classification of genomic data during testing |  |
| 1. Limitations of testing, e.g., what types of mutations are not detected by tests |  |
| **Ethical, legal, and social implications (ELSI) of genetic/genomic testing** |  |
| 1. Ethical implications, e.g., incidental findings, variants of uncertain/unknown significance*, etc. |  |
| 1. Legal implications, e.g., data sharing, privacy, confidentiality, insurance, etc. |  |
| 1. Psychosocial implications, e.g., family communication of information, reproductive options, linking patients to support groups, etc. |  |
| **Other (please specify)……………………** |  |

#### Supplementary Figure 1. Number of respondents who had previously learnt about none, some or all topics within each category of genomics education^1^


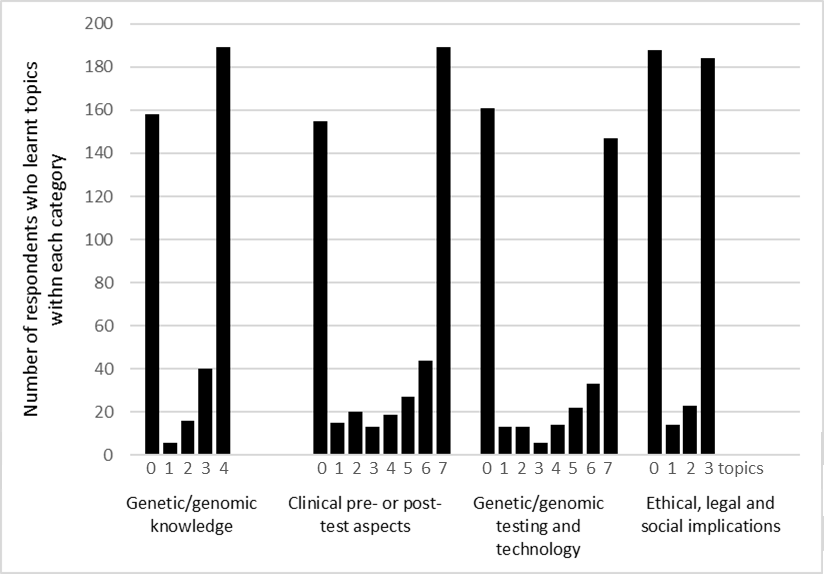


^1^ **Supplementary Table 5** lists topics within each category. Respondents could indicate having previously learnt no topics within a category (‘0’) or up to a maximum of four topics for ‘Genetic/genomic knowledge’, up to seven topics for ‘Clinical pre- or post-test aspects’ and ‘Genetic/genomic testing and technology’, and up to three topics for ‘Ethical, legal and social implications’.
